# Effects of In-Situ Reaction, Extrusion Ratio and CeO_2_ on the Performance of Al-Ti-C-(Ce) Grain Refiners for Refining Pure Aluminum Grains

**DOI:** 10.3390/ma16124481

**Published:** 2023-06-20

**Authors:** Qianwen Bi, Xiaoxu Luo, Lu Guo, Xiaoqing Zuo, Bei Huang, Jianhong Yi, Yun Zhou

**Affiliations:** 1 Faculty of Material Science and Engineering, Kunming University of Science & Technology, Kunming 650093, China; biqianwen_hui7@163.com (Q.B.);; 2 Faculty of Foreign Language and Culture, Kunming University of Science & Technology, Kunming 650093, China

**Keywords:** Al-Ti-C-(Ce) grain refiner, in-situ reaction, hot extrusion, second phase, grain-refining performance

## Abstract

Al-Ti-C-(Ce) grain refiners were prepared by combining in-situ reaction, hot extrusion, and adding CeO_2_. The effects of second phase TiC particle size and distribution, extrusion ratio, and Ce addition on the grain-refining performance of grain refiners were investigated. The results show that about 10 nm TiC particles are dispersed on the surface and inside of 100–200 nm Ti particles by in-situ reaction. The Al-Ti-C grain refiners, which are made, by hot extrusion, of a mixture of in-situ reaction Ti/TiC composite powder and Al powder, increase the effective nucleation phase of α-Al and hinder grain growth due to the fine and dispersed TiC; this results in the average size of pure aluminum grains to decrease from 1912.4 μm to 504.8 μm (adding 1 wt.% Al-Ti-C grain refiner). Additionally, with the increase of the extrusion ratio from 13 to 30, the average size of pure aluminum grains decreases further to 470.8 μm. This is because the micropores in the matrix of grain refiners are reduced, and the nano-TiC aggregates are dispersed with the fragmentation of Ti particles, resulting in a sufficient Al-Ti reaction and an enhanced nucleation effect of nano-TiC. Furthermore, Al-Ti-C-Ce grain refiners were prepared by adding CeO_2_. Under the conditions of holding for 3–5 min and adding a 5.5 wt.% Al-Ti-C-Ce grain refiner, the average size of pure aluminum grains is reduced to 48.4–48.8 μm. The reason for the excellent grain-refining and good anti-fading performance of the Al-Ti-C-Ce grain refiner is presumedly related to the Ti_2_Al_20_Ce rare earth phases and [Ce] atoms, which hinder agglomeration, precipitation, and dissolution of the TiC and TiAl_3_ particles.

## 1. Introduction

Aluminum and its alloys have the advantages of low density, good specific strength, and ductility, which are widely used in automobile, marine, aerospace, and other fields [1]. With the development of industries, the mechanical characteristics of aluminum and its alloys are subject to increasingly demanding standards. Fine grain strengthening, as one of the important mechanisms to improve the mechanical properties of aluminum products, can simultaneously improve the strength, toughness, and ductility of materials [2]. For example, Lei et al. [3] explored the effects of Sc and Zr on the grain refinement of aluminum and aluminum alloy. Vatansever et al. [4] used ultrasonic melt treatment to make α-Al phase of the dendrites smaller and improved the mechanical properties of the material. Among many grain-refining methods of aluminum and its alloys, the addition of grain refiner is simple and effective. Especially, Al-Ti-C grain refiners can immunize against the poisoning effect of alloying elements [5,6] and have therefore been widely investigated.

According to the classical duplex nucleation theory [7,8,9,10], the finer the size and more dispersed the distribution of the second phase in the Al-Ti-C grain refiner, the larger the number of effective nucleation phases of TiAl_3_ coated on TiC particles, and thus the better the refining performance [11]. Therefore, various researches mainly focus on strategies for controlling the size and distribution of the second phase in the grain refiners to increase the number of effective nucleated phases and to enable the smaller TiC particles to pinning grain boundaries more efficiently.

High-energy ball milling can prepare the in-situ reaction or ball milling loaded second phase particles with controlled size, dispersion, and elemental ratios, and is therefore used to manufacture grain refiners for aluminum and aluminum alloys. Liu et al. [12] prepared 5–30 nm TiC particles by a planetary high-energy ball mill with Ti powder and graphite powder, and the grain size of α-Al was reduced from 680 μm to 95 μm. Liu et al. [13] prepared Al/TiC and Al/TiB_2_ master alloys with evenly dispersed nano-TiC and TiB_2_ particles inside Al powder by a ball milling method and combustion synthesis reaction, and added them into the A1-Cu-Mg-Si alloy melt by stirring casting. The result showed that a remarkable grain refinement effect was obtained.

In addition, hot extrusion is also a common preparation method for obtaining PM (Powder metallurgy) grain refiners with fine and dispersed phases. Xu et al. [14] found that hot extrusion was effective at dispersing and refining aggregates in the grain refiner matrix, thereby increasing the number of nucleated particles and improving the refining effect. Zhao et al. [15] prepared Al-Ti-B grain refiners and found that a large number of fragmented TiAl_3_ particles were around TiAl_3_ particles after hot extrusion. Subsequent refinement experiments revealed that the grain refiner had a better refinement effect on industrial pure aluminum than the commercial Al-Ti-B grain refiners. In summary, hot extrusion has an important role in improving the refining performance of grain refiners.

Rare earth elements play a role in the modification and refinement of aluminum and aluminum alloys, and also improve the anti-fading performance. Zhao et al. [16] investigated the second phases of Al-3Ti-0.2C-1RE grain refiner and found that rare earth elements could be adsorbed onto the surface of TiAl_3_ to form a Ti_2_Al_20_RE phase, which hindered the growth of TiAl_3_, the formation of Al_4_C_3_, and the agglomeration of TiC. Ding et al. [17] prepared Al-Ti-C-Ce grain refiners and found that CeO_2_ promoted the formation of TiC through the phase analysis of a quenching experiment. Xu et al. [18] confirmed the contribution of the Ti_2_Al_20_Re phases on the anti-fading performance through the refinement experiments of Al-Ti-C-Re grain refiners. In addition, rare earth elements as a surfactant can improve the fluidity of melt and hinder the agglomeration and sedimentation of second phase particles [16,19].

Currently, studies prepare grain refiners with dispersed fine second phases by a single method, and there are few reports on Al-Ti-C-(Ce) grain refiners by a combined process of in-situ reaction, hot extrusion, and rare earth addition. In order to obtain Al-Ti-C grain refiners with excellent grain fining effect, Al-Ti-C grain refiners were first prepared by in-situ reaction and hot extrusion. The effects of TiC particle size and distribution, hot extrusion, and matrix microstructure density on the refinement of pure aluminum grains were investigated. On this basis, the effects of Ce element content and distribution on grain-refining and the anti-fading performance of Al-Ti-C-Ce grain refiners were investigated. This work will provide a useful reference for the research and development of Al-Ti-C/Al-Ti-C-Ce grain refiners with both excellent grain-refining and good anti-fading performance.

## 2. Experimental Procedure

### 2.1. Preparation of Ti/TiC Composite Powder and Al-Ti-C-(Ce) Grain Refiners

**Figure 1:**
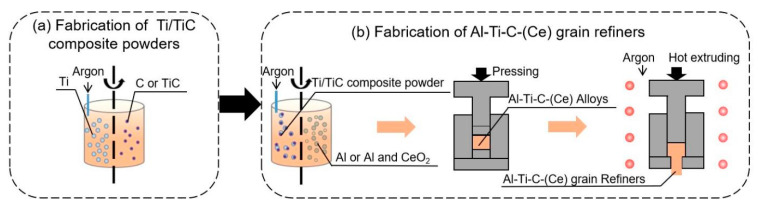
Flow diagram for the preparation of Ti/TiC composite powder and Al-Ti-C-(Ce) grain refiners: (**a**) fabrication of ball milling loaded and in-situ reaction Ti/TiC composite powder, (**b**) fabrication of Al-Ti-C-(Ce) grain refiners.

Figure 1 presents the flow diagram for the preparation of Ti/TiC composite powder and Al-Ti-C-(Ce) grain refiners. Table 1 presents the preparation parameters of composite powder and grain refiners. As shown in Figure 1a and Table 1, ball milling loaded and in-situ reaction Ti/TiC composite powder were prepared by ball milling, respectively. In the preparation of ball milling loaded Ti/TiC composite powder, 20 μm Ti powder and 200 nm TiC powder with a mass ratio of 10:1 were added into the ball mill tank, and 1 wt.% stearic acid (CH_3_(CH_2_)_16_COOH) was added as the process control agent to avoid sticking powders to tanks and balls, with a ball material ratio of 10:1, for 4 h under an argon atmosphere to prevent oxidation. In the preparation of in-situ reaction Ti/TiC composite powder, 20 μm Ti powder and 5 μm C powder with a mass ratio of 3.4:1 were added into a ball mill tank. The ball material ratio was 20:1, and the rotation speed was 300 rpm for 8–15 h under an argon atmosphere.

As shown in Figure 1b and Table 1, Al-Ti-C grain refiners were prepared as follows: the ball milling loaded or in-situ reaction Ti/TiC composite powder and the 10 μm Al powder were added into a ball mill tank with a mass ratio of 91.2:8.8, and mixing (0.5 wt.% stearic acid was added as the process control agent; the ball material ratio was 5:1, the rotation speed was 150 rpm for 1 h under an argon atmosphere), cold pressing (500 MPa pressure densifies the powders) and hot extrusion (extrusion ratios of 13:1, 20:1, and 30:1, after holding at 773 K under argon for 30 min, make the metallurgical bond between powders closer) were performed. In the preparation process for Al-Ti-C-Ce grain refiners, the 8.8 wt.% in-situ reaction Ti/TiC composite powder, 0.25 wt.%, 0.5 wt.%, 0.75 wt.%, 1.0 wt.%, 2.0 wt.% 1–3 μm CeO_2_ powder, and 10 μm Al powder were added into a ball mill tank, and the Al-Ti-C-Ce grain refiners were prepared by mixing (same as above), cold pressing (same as above), and hot extrusion (extrusion ratio was performed at 30:1, after holding at 773 K under an argon atmosphere for 30 min).

### 2.2. Refinement Experiment

**Figure 2:**
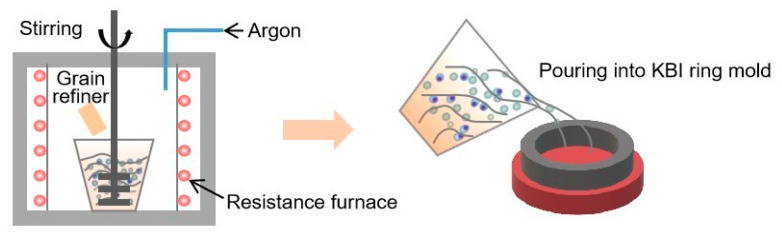
KBI ring model experiment was used to test the refining performance of pure aluminum grains.

The KBI ring mold [20] and the grain refiners were preheated to 473 and 383 K, respectively. Aluminum ingot (≥99.7%) was placed in a graphite crucible in an argon atmosphere and melted at 1003 K in a resistance furnace. To remove the oxides and slags from the melt surface, the molten aluminum was then degassed using a 1 wt.% C_2_Cl_6_ refining agent and kept for 5 min. As shown in Figure 2, the preheated grain refiner was added to the pure aluminum melt, which was stirred continuously at 700 rpm for 90 s by TC4 titanium alloy stirring paddle and held for 1–15 min, and then poured into the preheated KBI ring mold to control the grain-refining process and verify the refining effect.

### 2.3. Characterization

The Ti/TiC composite powder were observed by a field emission scanning electron microscope (FSEM, Hitachi SU8010, Tokyo, Japan), high-resolution transmission electron microscopy (HRTEM, Tecnai G2 TF30, FEI, Amsterdam, The Netherlands), and energy disperse spectroscopy (EDS, Cambridge, UK). The in-situ reaction Ti/TiC composite powder phase constituents were analyzed by X-ray diffraction (XRD, D/MAX 2550, Tokyo, Japan), and the characteristic peaks of TiC and C were further confirmed by LabRAM HR Evolution. The microstructures of grain refiners were observed by tungsten filament scanning electron microscopy (SEM, VEGA3 SBH, TESCAN, Jena, Germany), and EDS.

The grain size of pure aluminum was measured using the intercept method. An inspection line was drawn in the microstructure diagram of pure aluminum taken by the Shenhong magnifying glass, and the number of grains passed through the inspection line was counted. To ensure data accuracy, three regions were randomly selected for each sample to measure grain size and to take the average value, which can be estimated by Equation (1):

(1) I=L/NG

where *I* is the average size of pure aluminum grains (μm), *L* is the inspection line length (μm), *N* is the number of intersections between the inspection line and the grain boundary, and *G* is the magnification.

## 3. Results and Discussion

### 3.1. Ball Milling Loaded and In-Situ Reaction Ti/TiC Composite Powder

#### 3.1.1. Size and Distribution of Ball Milling Loaded Ti/TiC Composite Powder

**Figure 3:**
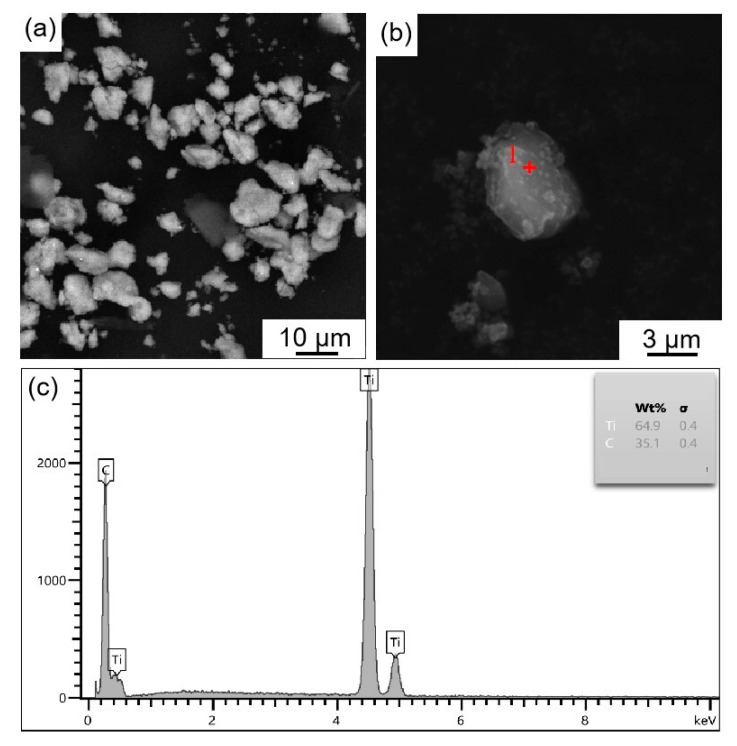
Microstructure and EDS images of ball milling loaded Ti/TiC composite powder: (**a**) ball milling loaded Ti/TiC composite powder (1#), (**b**) ball milling loaded Ti/TiC single particle, (**c**) point 1 EDS analysis.

**Figure 4:**
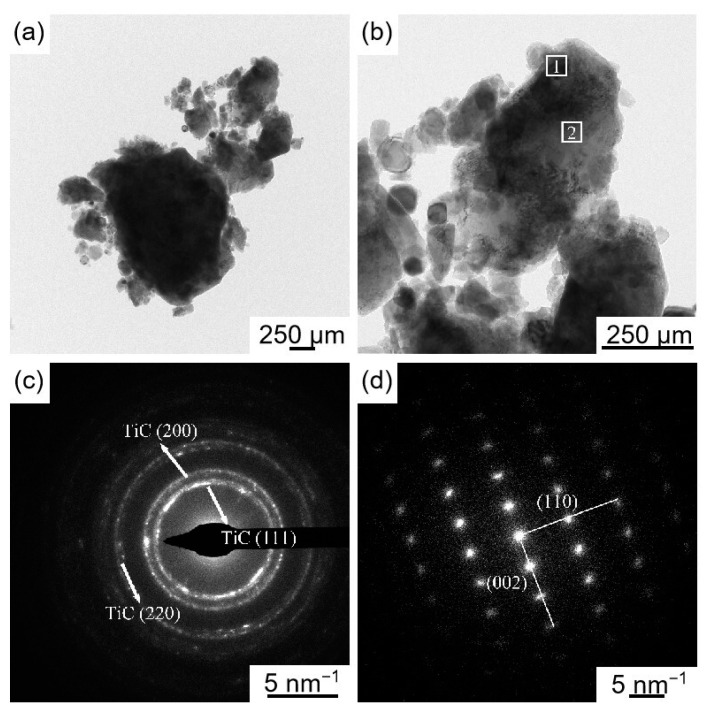
TEM and SAED images of ball milling loaded Ti/TiC composite powder: (**a**) ball milling loaded Ti/TiC composite powder (1#), (**b**) ball milling Ti/TiC single particle, (**c**) analysis of SAED patterns in region 1, (**d**) analysis of SAED patterns in region 2.

Figure 3 shows the microstructure and EDS images of ball milling loaded Ti/TiC composite powder. As seen in Figure 3a, after ball milling for 4 h, the size of Ti powder was reduced from the initial 20 μm to 1–10 μm due to the action of fracture-welding [21] in ball milling. Many small particles were attached to the surface of Ti powder particles (Figure 3b). The point scanning analysis of a bright particle (point 1) revealed that the constituent elements of the particles attached to the surface of Ti powder are Ti and C (Figure 3c), which are presumed to be TiC particles. Figure 4 shows the TEM and SAED images of ball milling loaded Ti/TiC composite powder. As shown in Figure 4a,b, the margins of Ti particles showed dark areas. The diffraction pattern of dark field region 1 was indexed (Figure 4c), and the diffraction ring radii were measured to be 4.01 nm^−1^, 4.63 nm^−1^, and 6.59 nm^−1^, corresponding to (111), (200), and (220) crystal planes of TiC, respectively. The diffraction pattern in the light field region 2 was indexed (Figure 4d), and the crystal plane spacings of the two spots at the right angle were measured to be 0.1431 nm and 0.2347 nm, which corresponds to the crystal planes of Ti (110) and (002), respectively. In summary, Ti/TiC composite powder with TiC particle sizes of about 1–3 μm are successfully loaded onto micron Ti powder through a ball milling loading process.

#### 3.1.2. Size and Distribution of In-Situ Reaction Ti/TiC Composite Powder

**Figure 5:**
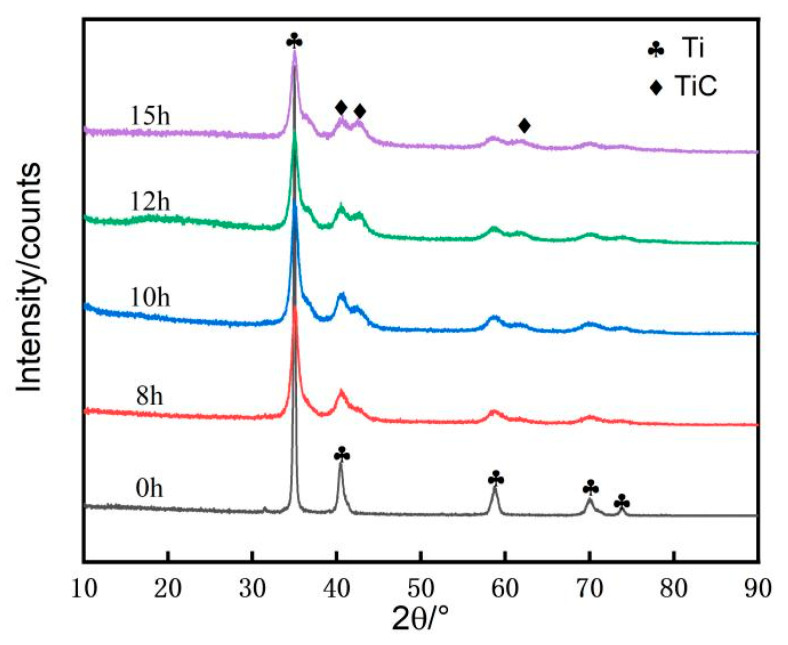
The XRD pattern of in-situ reaction Ti/TiC composite powder in high-energy ball milling for 0–15 h.

Figure 5 shows the XRD pattern of in-situ reaction Ti/TiC composite powder in high-energy ball milling for 0–15 h. As seen in Figure 5, after 8 h of high-energy ball milling, the diffraction peak of Ti gradually broadened and shifted, but the diffraction peak of TiC was not evident, showing that the stress generated by high-energy ball milling or the solid solution C in Ti crystals cause a change in the Ti lattice constant. After 10 h of high-energy ball milling, diffraction peaks of TiC appeared obviously and the bottom of the peak broadened, indicating that TiC is created when C powder adheres to Ti particles during high-energy ball milling, resulting in TiC diffraction peaks superimposing on the original Ti diffraction peaks.

**Figure 6:**
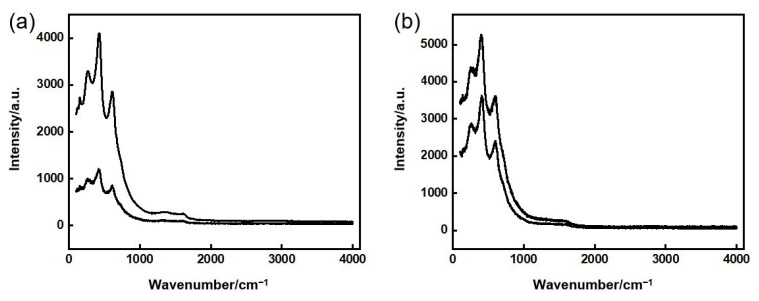
The Raman spectrums of in-situ reaction Ti/TiC composite powder: (**a**) high-energy ball milling for 12 h, (**b**) high-energy ball milling for 15 h.

To further determine whether the reaction of C powder with Ti powder was complete after the in-situ reaction, Figure 6 shows the Raman spectrums of in-situ reaction Ti/TiC composite powder after 12 h and 15 h of high-energy ball milling. After 12 h of high-energy ball milling, the TiC characteristic peaks were observed at 260 cm^−1^, 420 cm^−1^, and 605 cm^−1^, but the C characteristic peaks were observed after 1000 cm^−1^ (Figure 6a), indicating that the reaction between C powder and Ti powder is incomplete after 12 h of high-energy ball milling. Meanwhile, after 15 h of high-energy ball milling, the characteristic peak of TiC was obvious and the characteristic peak of C was weakened (Figure 6b), indicating that TiC is in-situ-generation sufficient and C powder is basically completely reacted, but there is still a small amount of residue.

**Figure 7:**
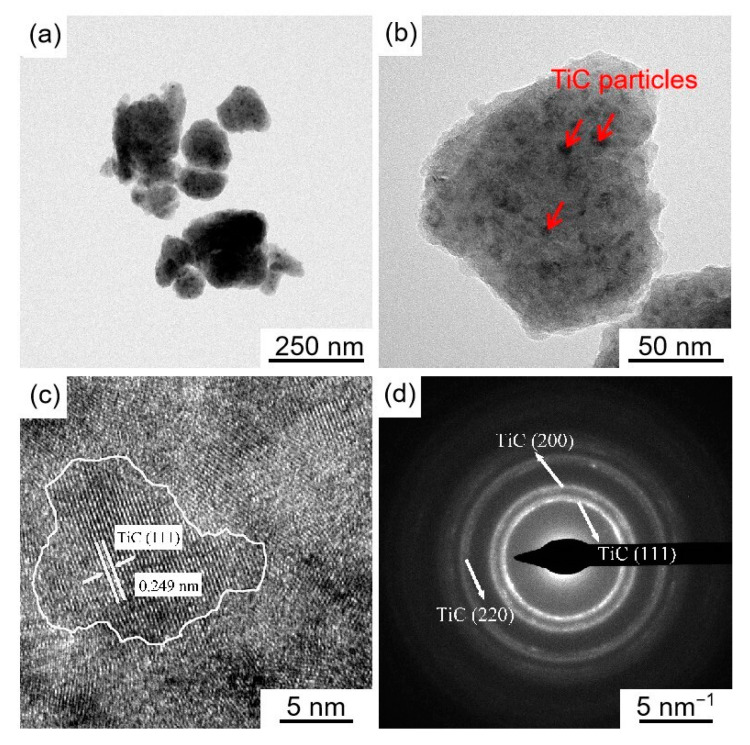
TEM and SAED images of in-situ reaction Ti/TiC composite powder: (**a**) in-situ reaction Ti/TiC composite powder (2#), (**b**) in-situ reaction Ti/TiC single particle, (**c**) analysis of HRTEM, (**d**) analysis of SAED patterns.

Figure 7 shows TEM and SEAD images of in-situ reaction Ti/TiC composite powder. As seen in Figure 7a, the size of Ti powder after high-energy ball milling was reduced from the initial 20 μm to 100–200 nm. Unlike the ball milling loaded composite powder, dark particles of about 10 nm were produced by in-situ reaction and evenly distributed on the surface and inside of the submicron Ti particles (Figure 7b). The crystalline spacing of the dark particles in the selected area was 0.249 nm (Figure 7c), which was close to the TiC (100) crystalline spacing. The diffraction pattern of the selected lattice stripe image was indexed with diffraction ring radii corresponding to the (111), (200), and (220) crystal planes of TiC, respectively (Figure 7d). Therefore, it was finally determined that in-situ reactions yielded TiC particles of about 10 nm.

**Figure 8:**
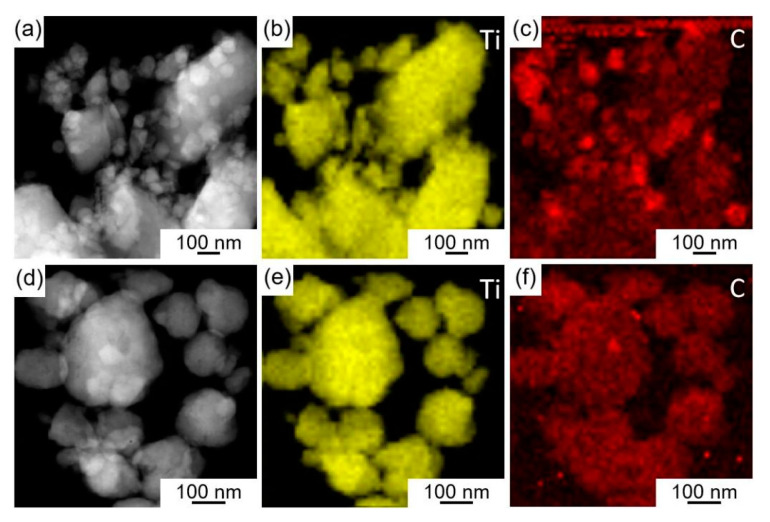
STEM and EDS images with Ti and C elements of ball milling loaded and in-situ reaction Ti/TiC composite powder: (**a**–**c**) ball milling loaded Ti/TiC composite powder (1#), (**d**–**f**) in-situ reaction Ti/TiC composite powder (2#).

Figure 8 depicts the STEM and EDS images with Ti and C elements of ball milling loaded and in-situ reaction Ti/TiC composite powder. Unlike the evident C element aggregated on the surface of Ti particles in the ball milling loaded Ti/TiC composite powder (1#) (Figure 8a–c), the C elements in the Ti particles of the in-situ reaction Ti/TiC composite powder (2#) (Figure 8d–f) exhibited a slight agglomeration phenomenon, but the overall distribution was even.

#### 3.1.3. Influence of Ti/TiC Composite Powder on the Grain-Refining Performance of Al-Ti-C Grain Refiners

**Figure 9:**
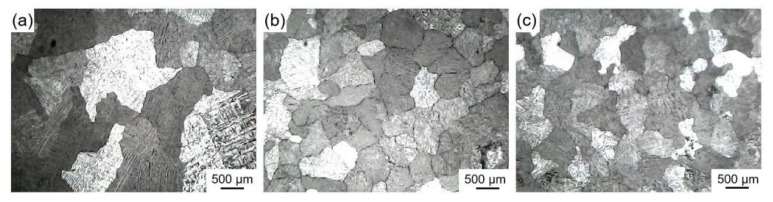
Microstructure of pure aluminum before and after being refined by different Al-Ti-C grain refiners (add 1 wt.%): (**a**) no grain refiner, (**b**) ball milling loaded Al-Ti-C grain refiner (3#), (**c**) in-situ reaction Al-Ti-C grain refiner (4#).

Figure 9 shows the microstructure of pure aluminum before and after being refined by ball milling loaded and in-situ reaction Al-Ti-C grain refiners. Table 2 shows the average size of corresponding pure aluminum grains before and after being refined by different grain refiners. According to Figure 9 and Table 2, the average size of pure aluminum grains without a grain refiner was 1912.4 μm, and the average size of pure aluminum grains was, respectively, refined to 580.2 μm and 504.8 μm by adding a 1 wt.% of ball mill load (3#) and an in-situ reaction Al-Ti-C grain refiner (4#). This suggests that the addition of TiC particles effectively enhances the refining effect of the grain refiners. Meanwhile, compared with ball milling loaded, the refining performance of the in-situ reaction Al-Ti-C grain refiner was increased by 13%. The improved refining performance is primarily attributable to the formation of more TiAl_3_ particles as a result of the uniform distribution of submicron Ti particles produced by the in-situ reaction of Ti/TiC composite powder and aluminum melt. At the same time, the evenly distributed nano-TiC particles on the surface and inside the Ti powder gradually separate, and Ti-rich zones form around the nano-TiC after the high-temperature dissolving of TiAl_3_, resulting in the generation of TiAl_3_ thin layers on the surface of the nano-TiC particles and an increase in the effective nucleation phase [11]. Furthermore, it is speculated that the nano-TiC and nano-Al_2_O_3_ form a dense particle shell layer at the α-Al grain boundary, inhibiting the diffusion of solute atom into the solid/liquid interface and the grain growth [13].

### 3.2. Influence of Extrusion Ratio on Element Distribution and Grain-Refining Performance of Al-Ti-C Grain Refiners

**Figure 10:**
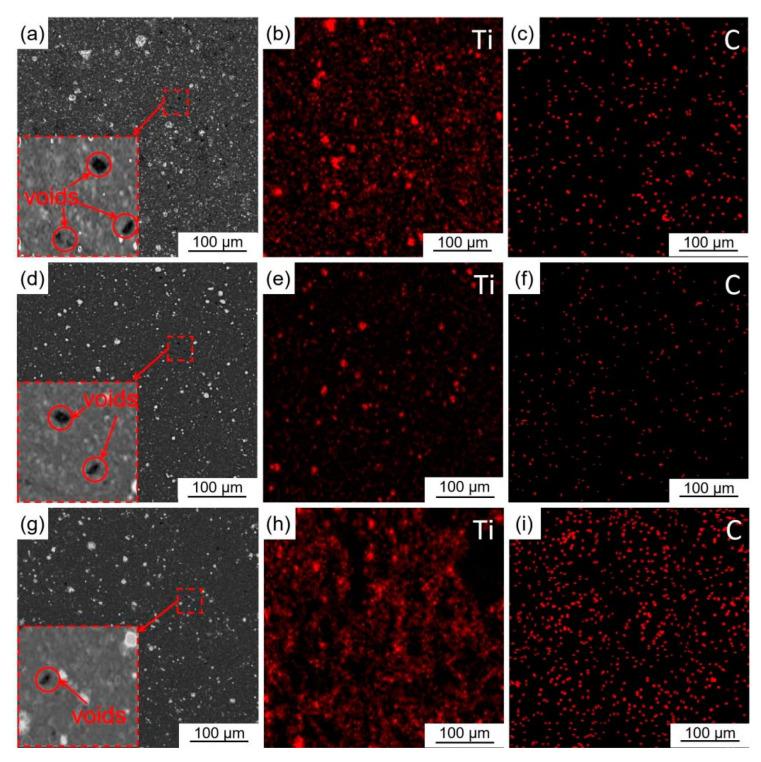
Microstructure and EDS images of in-situ reaction Al-Ti-C grain refiners with various extrusion ratios: (**a**–**c**) extrusion ratio 13 grain refiner (4#), (**d**–**f**) extrusion ratio 20 grain refiner (5#), (**g**–**i**) extrusion ratio 30 grain refiner (6#).

Figure 10 shows the microstructure and EDS images of Al-Ti-C grain refiners with various extrusion ratios. According to Figure 10a,d,g, almost no obvious micro-pores and cracks were observed in the microstructure of Al-Ti-C grain refiner (6#) when the extrusion ratio increased from 13 to 30, indicating that the increasing extrusion ratio causes a tighter metallurgical bonding and a higher matrix microstructure density. According to Figure 10b,e,h, the Ti element agglomeration phenomenon reduced, indicating that increasing the extrusion ratio breaks up the Ti particle agglomerates and disperses them uniformly in the matrix. According to Figure 10c,f,i, the distribution points of the C element increased, indicating that the nano-TiC particles distributed on Ti particles are also evenly dispersed with the fragmentation of Ti aggregates.

**Figure 11:**
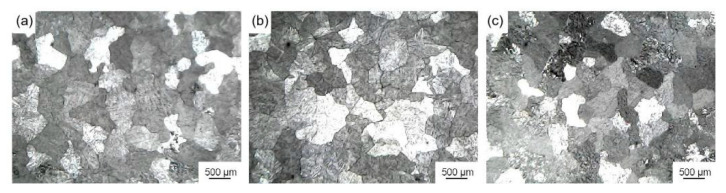
Microstructure of pure aluminum after being refined by Al-Ti-C grain refiners with various extrusion ratios (add 1 wt.%): (**a**) extrusion ratio 13 grain refiner (4#), (**b**) extrusion ratio 20 grain refiner (5#), (**c**) extrusion ratio 30 grain refiner (6#).

Figure 11 shows the microstructure of pure aluminum after being refined by extrusion ratios of 13, 20, and 30 Al-Ti-C grain refiners. Table 3 shows the average size of corresponding pure aluminum grains after being refined by different grain refiners. As shown in Figure 11 and Table 3, by the addition of 1 wt.% extrusion ratio 13, 20, and 30 Al-Ti-C grain refiners (4#–6#) to pure aluminum melt, the average size of pure aluminum grains were refined to 504.8 μm, 492.4 μm, and 470.8 μm, respectively. The refining performance of extrusion ratio 30 Al-Ti-C grain refiner increased by 12% compared with extrusion ratio 13. According to the analysis in Figure 10, the increasing extrusion ratio could densify the matrix microstructure, break Ti aggregates [15], disperse nano-TiC particles, and make the Al-Ti reaction adequate. Meanwhile, the melting temperature increased with the Al-Ti reaction, improving the wettability between TiC and Al melt, while also causing the rapid dissolution of TiAl_3_ particles to form a Ti-rich zone and thus promoting the nucleation ability of TiC [11]. In addition, the hot extrusion causes grain refinement by producing fragmented nano-Al_2_O_3_, which inhibited the growth of α-Al grains at the boundaries [13].

### 3.3. Influence of CeO_2_ on the Grain-Refining Performance of Al-Ti-C-(Ce) Grain Refiners

#### 3.3.1. Influence of Al-Ti-C Grain Refiner Content on Grain-Refining Performance

**Figure 12:**
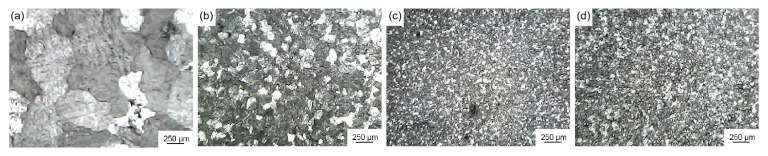
Microstructure of pure aluminum after being refined by extrusion ratio 30 Al-Ti-C grain refiner with different addition amounts: (**a**) 1.0 wt.% grain refiner (6#), (**b**) 3.0 wt.% grain refiner (6#), (**c**) 5.5 wt.% grain refiner (6#), (**d**) 6.0 wt.% grain refiner (6#).

Figure 12 shows the microstructure of pure aluminum after being refined with 1.0 wt.%, 3.0 wt.%, 5.5 wt.%, and 6.0 wt.% extrusion ratio 30 Al-Ti-C grain refiner. Table 4 shows the average size of pure aluminum grains after being refined by grain refiners. As shown in Figure 12 and Table 4, the average size of corresponding pure aluminum grains reduced to 504.8 μm and 124.6 μm at 1.0 wt.% and 3.0 wt.% with the addition of grain refiner (6#), respectively. The best grain-refining effect was achieved at 5.5 wt.% of grain refiner addition, and the average size of pure aluminum grains was refined to 52.4 μm. As the amount of grain refiner increases, the reaction product TiAl_3_ and the effective nucleation phase may both rise. With the addition of 6.0 wt.% of the grain refiner, the average size of pure aluminum grains increased to 54.6 μm. This is presumably related to the decrease of effective nucleation phases caused by the agglomeration of nano-TiC particles in the Al melt.

#### 3.3.2. Influence of CeO_2_ Content on Element Distribution and Grain-Refining Performance of Al-Ti-C-(Ce) Grain Refiners

**Figure 13:**
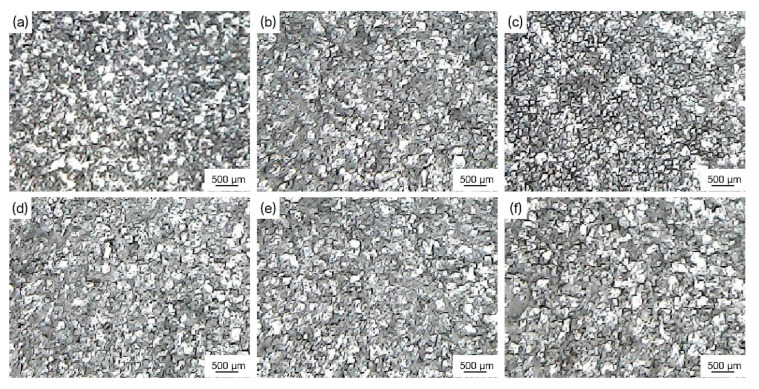
Microstructure of pure aluminum after being refined by Al-Ti-C-Ce grain refiners (add 5.5 wt.%) with different CeO_2_ content: (**a**) 0 wt.% CeO_2_ grain refiner (6#), (**b**) 0.25 wt.% CeO_2_ grain refiner (7#), (**c**) 0.50 wt.% CeO_2_ grain refiner (8#), (**d**) 0.75 wt.% CeO_2_ grain refiner (9#), (**e**) 1.0 wt.% CeO_2_ grain refiner (10#), (**f**) 2.0 wt.% CeO_2_ grain refiner (11#).

Figure 13 shows the microstructure of pure aluminum after being refined with Al-Ti-C-(Ce) grain refiners (adding 5.5 wt.%) at 0.5 wt.% and 2.0 wt.% CeO_2_. Table 5 shows the average size of corresponding pure aluminum grains after being refined by different grain refiners (add 5.5 wt.%). As shown in Figure 13 and Table 5, Al-Ti-C-Ce grain refiner (7#) is more effective than Al-Ti-C grain refiner (6#). This result could be attributed to the following reasons. Firstly, the [Ce] atoms generated by the reaction of CeO_2_ with the Al melt are reacted with the TiAl_3_ surfaces to form the Ti_2_Al_20_Ce core-shell structured rare earth phase, which has a better nucleation ability and inhibits the decomposition of TiAl_3_ and the growth of crystal nuclei [22], thus increasing the number of effective nucleation phases. Secondly, the [Ce] atoms with strong chemical activity improve the wettability and dispersibility of TiC in Al melts. Furthermore, [Ce] atoms segregate around the α-Al grains [16] and inhibit grain growth. Finally, the TiAl_3_ and TiC particles uniformly disperse in the melt under the combined effect of the mechanical stirring and the tumbling phenomenon generated by the reaction of CeO_2_ with the residual C powder, which hinders the agglomeration and sedimentation of the second phase.

**Figure 14:**
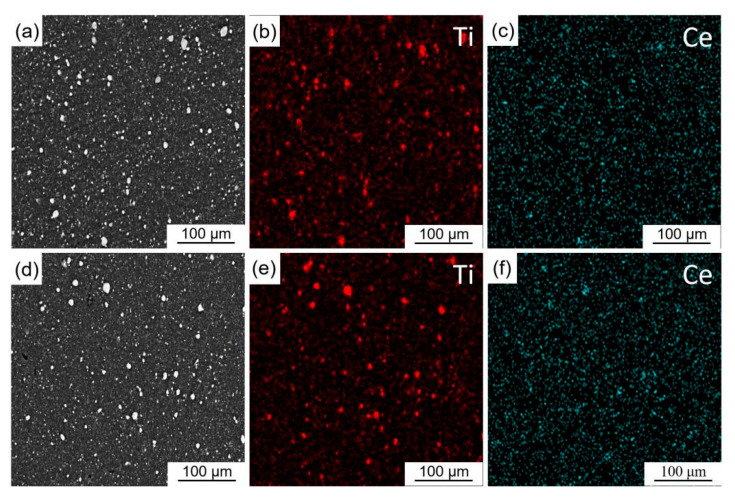
Microstructure and EDS images of Al-Ti-C-Ce grain refiners with different CeO_2_ contents: (**a**–**c**) 0.50 wt.% CeO_2_ grain refiner (8#), (**d**–**f**) 2.0 wt.% CeO_2_ grain refiner (11#).

In addition, as shown in Figure 13c and Table 5, 0.5 wt.% CeO_2_ Al-Ti-C-Ce grain refiner (8#) has the best refining performance, and the average size of pure aluminum grains decreased to 48.4 μm. With the increase of CeO_2_ addition, the refining effect of Al-Ti-C-Ce grain refiner (9#–11#) remains essentially unchanged (Figure 13d–f). To further analyze these reasons, Figure 14 shows the microstructure and EDS images of 0.5 wt.% and 2.0 wt.% CeO_2_ Al-Ti-C-Ce grain refiners. As shown in Figure 14, the Al-Ti-C-Ce grain refiner was prepared with the addition of 0.5 wt.% CeO_2_, and the element Ce was evenly distributed in the matrix of the grain refiner. When the element Ce content in Al melt was sufficient (Figure 14c), some [Ce] atoms were enriched on the surface of nano-TiC and form a protective layer, which hindered TiC reaction to form Al_4_C_3_ [16] and TiC aggregation. However, as the content of CeO_2_ in the grain refiner increased, the fine aggregates of element Ce increased, and thus the formation of Ti_2_Al_20_Ce phases and [Ce] atom involved in grain refinement reached saturation as the CeO_2_ content in the Al melt increased (Figure 14f), resulting in the grain-refining performance to be no longer enhanced.

#### 3.3.3. Influence of CeO_2_ on Grain-Refining and Anti-Fading Performance of Al-Ti-C-(Ce) Grain Refiners with Different Holding Time

**Figure 15:**
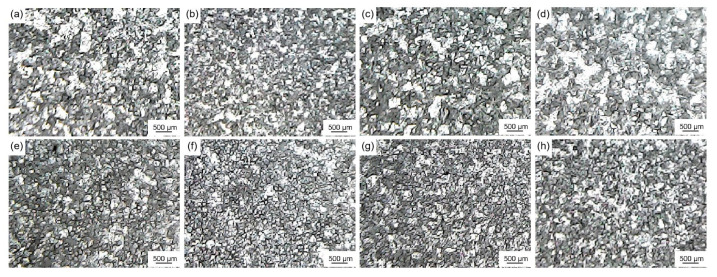
Microstructure of pure aluminum after being refined by Al-Ti-C and Al-Ti-C-Ce grain refiners (add 5.5 wt.%) with different holding times: (**a**–**d**) by Al-Ti-C grain refiner (6#) with 1, 3, 5, and 10 min, (**e**–**h**) by Al-Ti-C-Ce grain refiner (8#) with 1, 3, 5, and 10 min.

Figure 15 shows the microstructure of pure aluminum after being refined by Al-Ti-C and Al-Ti-C-Ce grain refiners with holding for 1, 3, 5, and 10 min, respectively. Table 6 shows the average size of corresponding pure aluminum grains after being refined by different grain refiners. As shown in Figure 15 and Table 6, the addition of 5.5 wt.% Al-Ti-C grain refiner (6#) and Al-Ti-C-Ce grain refiner (8#) to pure aluminum melt for 1 min, the average size of pure aluminum grains was refined to 62.5 μm and 54.6 μm, respectively. After holding for 3 min, the average size of pure aluminum grains was 52.4 μm and 48.4 μm, respectively. After holding for 5 min, the Al-Ti-C grain refiner showed a decline in grain-refining and the average size of pure aluminum grains increased to 64.8 μm. This is caused by the agglomeration and sedimentation of the Al_4_C_3_ produced by the reaction of the second phase TiC and the massive dissolution of the thin TiAl_3_ layer coated on the TiC surface. However, the Al-Ti-C-Ce grain refiner did not show the phenomenon of grain-refining fading. This result is related to the Ti_2_Al_20_Ce phase and [Ce] atoms coated on the surface of TiAl_3_ and TiC, thus prolonging the nucleation time of the second phase in Al melt [16,23]. With severe grain-refining fading, the average size of pure aluminum grains increased to 116.4 μm and 68.4 μm after holding for 10 min. In conclusion, the Al-Ti-C-Ce grain refiner has a better grain refinement and anti-fading performance than the Al-Ti-C grain refiner.

#### 3.3.4. Analysis of the Refinement Mechanism of Al-Ti-C-Ce Grain Refiner

In order to clarify the role of CeO_2_ in the grain-refining process of the Al-Ti-C-Ce grain refiner, the main reactions and standard Gibbs free energy changes in this system were determined. The possible reactions are shown in Equations (2)–(5). Table 7 shows the Gibbs free energy of the above reactions at the experimental temperature of 1003 K.

(2) $$\begin{array}{c} \mathrm{Ti}(s)+3\mathrm{Al}(l)=\mathrm{Ti}{\mathrm{Al}}_{3}\left(s\right)\\ {∆G}_{1}=-182.827+0.06377T\;(\mathrm{KJ}\cdot {\mathrm{mol}}^{-1}) \end{array}$$

(3) $$\begin{array}{c} CeO_{2}\left(s\right)+4C(s)=CeC_{2}(s)+2CO(g)\\ ∆G_{2}=1099.818-0.6217T\;(\mathrm{KJ}\cdot {\mathrm{mol}}^{-1}) \end{array}$$

(4) $$\begin{array}{c} CeC_{2}\left(s\right)+2Ti\left(s\right)=2TiC+[Ce]\\ ∆G_{3}=-621.178+0.22623T\;(\mathrm{KJ}\cdot {\mathrm{mol}}^{-1}) \end{array}$$

(5) $$\left[Ce\right]+2Ti{Al}_{3}\left(s\right)+14Al\left(l\right)={Ti}_{2}{Al}_{20}Ce(s)$$

**Figure 16:**
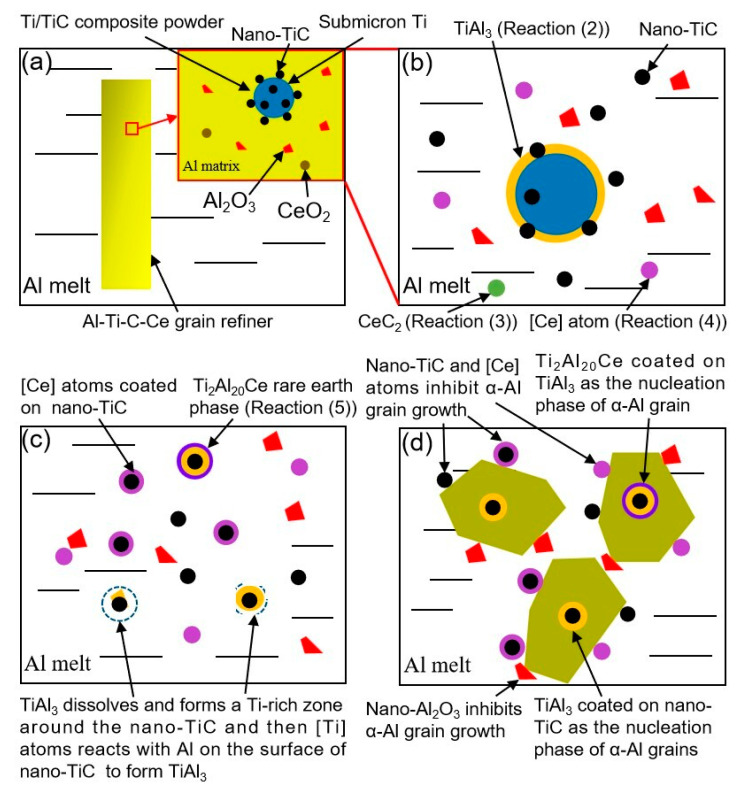
Schematic diagram of refining mechanism of the Al-Ti-C-Ce grain refiner: (**a**) schematic diagram of grain refiner and its local microstructure, (**b**) reactions of the grain refiner in Al melt to form TiAl_3_ and [Ce] atoms, (**c**) formation of Ti-rich zones and TiAl_3_ thin layers, adsorption of [Ce] atoms, and formation of Ti_2_Al_20_Ce rare earth phases, (**d**) α-Al grain nucleation and growth.

Figure 16 shows a schematic diagram of the refining mechanism of the Al-Ti-C-Ce grain refiner. After the addition of the Al-Ti-C-Ce grain refiner to the Al melt, Ti, CeO_2_, and Al_2_O_3_ submicron particles in the matrix are exposed after the surface Al is melted (Figure 16a). As can be seen from Table 7, ∆G_1_ < 0, with the Al-Ti reaction (2) proceeds, the TiAl_3_ particles form on the surface of submicron Ti particles (Figure 16b). As the grain refiner melts, the CeO_2_ particles are uniformly dispersed in the melt. ∆G_2_ > 0, although the experimental temperature does not reach the temperature required for reaction (3), the Al-Ti exothermic reaction (2) provides the heat required for the reaction, leading to the reaction (3) of CeO_2_ with residual C powder and the formation of CO gas and CeC_2_. This result is consistent with the study of Ding et al. [24]. CO gas tumbles in Al melt and further disperses CeC_2_, TiAl_3_, TiC, and Al_2_O_3_ particles in the melt, which prevents the agglomeration and precipitation of the second phase particles.

The instability of TiAl_3_ formed by the Al-Ti reaction (2) redissolves and generates [Ti] atoms at 1003 K [22,25]. These atoms then form Ti-rich zones around TiC and re-form thin layers of TiAl_3_ on the dispersed TiC surface (Figure 16c), which promotes α-Al nucleation [7,8,9,10] (Figure 16d). ∆G_3_ < 0, evenly distributed CeC_2_ particles react to generate TiC and the released [Ce] atoms (reaction (4)) (Figure 16b). [Ce] atoms adsorb on the surface of TiAl_3_, and the reaction (5) forms a core-shell structure of Ti_2_Al_20_Ce coated on TiAl_3_ [18] (Figure 16c), which inhibits the growth of TiAl_3_ phase crystal nuclei and promotes α-Al nucleation [16,23] (Figure 16d). In the meantime, part of the Ti_2_Al_20_Ce phase continuously releases [Ce] atoms [17] into the melt during the holding process. The [Ce] atoms cluster on the TiC surface (Figure 16c) to improve the wettability between TiC and melt Al, preventing the generation of Al_4_C_3_ from TiC [16]. Reaction (5) is only a speculative result, and the theoretical analysis of its reaction thermodynamics and kinetics needs further study, but the result that the reaction generated Ti_2_Al_20_Ce phase is consistent with the theories of Zhao et al. [16] and Ding et al. [24]. During the solidification phase, nano-TiC and nano-Al_2_O_3_ are pushed to the grain boundaries, which inhibits grain growth combined with [Ce] atoms (Figure 16d).

## 4. Conclusions

The rod-shaped Al-Ti-C-(Ce) grain refiners have a very fast refining response rate, which is suitable for the continuous casting production of aluminum, and a good refining effect can be achieved by sending the grain refiner into the flow channels between the stationary furnace and the casting machine in the wire feeder. The main findings of this paper are as follows:

(1) Ball milling loading formed TiC particles on the surface of Ti particles, while in-situ reaction generated TiC particles of about 10 nm that were evenly dispersed on the surface and inside of the Ti particles. Dispersed nano-TiC particles effectively improve nucleation efficiency and inhibit grain growth. Compared with ball milling loading, the refining performance of in-situ reaction Al-Ti-C grain refiner (1 wt.%) increased by 13%.
(2) With an increase of the extrusion ratio from 13 to 30, the matrix microstructure of the grain refiner became denser and the fine Ti agglomerates were broken, resulting in a more even distribution of nano-TiC particles and an increase of the Al-Ti reaction product TiAl_3_ during the grain-refining process; thus, the number of effective nucleation phases increased, and the refining performance of in-situ reaction Al-Ti-C grain refiner (1 wt.%) further increased by 12%.
(3) Al-Ti-C grain refiners containing 0.5 wt.% CeO_2_ were prepared by a combined in-situ reaction, hot extrusion, and rare earth addition. Under the conditions of 5.5 wt.% Al-Ti-C-Ce grain refiner addition and 3–5 min holding, the average grain size of pure aluminum was refined from 1912.4 μm to 48.4–48.8 μm and no refinement fade was observed, indicating its grain refinement performance is better than that of the Al-Ti-C grain refiner. The reason for this is presumably related to the Ti_2_Al_20_Ce rare earth phase and [Ce] atoms on the TiC and TiAl_3_ surfaces, which slow down the agglomeration and precipitation of the TiC and TiAl_3_ particles, increase the effective nucleation phase, and delay the nucleation failure of the second phase under the combined effect of CO gas tumbling.

## Figures and Tables

**Table 1:** Preparation parameters of Ti/TiC composite powder and Al-Ti-C-(Ce) grain refiners.

No.	Name	Composition of Powder				
		Al/wt.%	Ti/wt.%	TiC/wt.%	C/wt.%	CeO_2_/wt.%
1#	Ball milling loaded Ti/TiC composite powder	-	8.0	0.8	-	-
2#	In-situ reaction Ti/TiC composite powder	-	6.8	-	2.0	-
3#	Extrusion ratio 13 and ball milling loaded Al-Ti-C grain refiner	91.2	8.0	0.8	-	-
4#	Extrusion ratio 13 and in-situ reaction Al-Ti-C grain refiner	91.2	6.8	-	2.0	-
5#	Extrusion ratio 20 and in-situ reaction Al-Ti-C grain refiner	91.2	6.8	-	2.0	-
6#	Extrusion ratio 30 and in-situ reaction Al-Ti-C grain refiner	91.2	6.8	-	2.0	-
7#	Extrusion ratio 30, 0.25 wt.% CeO_2_ and in-situ reaction Al-Ti-C-Ce grain refiner	90.95	6.8	-	2.0	0.25
8#	Extrusion ratio 30, 0.50 wt.% CeO_2_ and in-situ reaction Al-Ti-C-Ce grain refiner	90.7	6.8	-	2.0	0.50
9#	Extrusion ratio 30, 0.75 wt.% CeO_2_ and in-situ reaction Al-Ti-C-Ce grain refiner	90.45	6.8	-	2.0	0.75
10#	Extrusion ratio 30, 1.0 wt.% CeO_2_ and in-situ reaction Al-Ti-C-Ce grain refiner	90.2	6.8	-	2.0	1.0
11#	Extrusion ratio 30, 2.0 wt.% CeO_2_ and in-situ reaction Al-Ti-C-Ce grain refiner	89.2	6.8	-	2.0	2.0

**Table 2:** Average size of pure aluminum grains before and after being refined by different Al-Ti-C grain refiners (add 1 wt.%).

Grain Refiner	Average Grain Size/μm
No Grain Refiner	1912.4
Ball Milling Loaded Al-Ti-C Grain Refiner (3#)	580.2
In-Situ Reaction Al-Ti-C Grain Refiner (4#)	504.8

**Table 3:** Average size of pure aluminum grains after being refined by Al-Ti-C grain refiners with different extrusion ratios (add 1 wt.%).

Grain Refiner	Extrusion Ratio	Average Grain Size/μm
Grain Refiner (4#)	13	504.8
Grain Refiner (5#)	20	492.4
Grain Refiner (6#)	30	470.8

**Table 4:** Average size of pure aluminum grains after being refined by extrusion ratio 30 Al-Ti-C grain refiner (6#) with different addition amounts.

Grain Refiner	Content of Grain Refiner/wt.%	Average Grain Size/μm
Grain Refiner (6#)	1.0	504.8
Grain Refiner (6#)	3.0	124.6
Grain Refiner (6#)	5.5	52.4
Grain Refiner (6#)	6.0	54.6

**Table 5:** Average size of pure aluminum grains after being refined by Al-Ti-C-Ce grain refiners (add 5.5 wt.%) with different CeO_2_ contents.

Grain Refiner	CeO_2_ Content in Grain Refiner/wt.%	Average Grain Size/μm
Grain Refiner (6#)	0	52.4
Grain Refiner (7#)	0.25	50.8
Grain Refiner (8#)	0.50	48.4
Grain Refiner (9#)	0.75	48.6
Grain Refiner (10#)	1.0	48.7
Grain Refiner (11#)	2.0	48.6

**Table 6:** Average size of pure aluminum grains after being refined by Al-Ti-C and Al-Ti-C-Ce grain refiners (add 5.5 wt.%) with different holding times.

Grain Refiner	CeO_2_ Content in Grain Refiner/wt.%	Holding Time/min	Average Grain Size/μm
Grain Refiner (6#)	0	1	62.5
Grain Refiner (6#)	0	3	52.4
Grain Refiner (6#)	0	5	64.8
Grain Refiner (6#)	0	10	116.4
Grain Refiner (8#)	0.50	1	54.6
Grain Refiner (8#)	0.50	3	48.4
Grain Refiner (8#)	0.50	5	48.8
Grain Refiner (8#)	0.50	10	68.4

**Table 7:** Gibbs free energy of the above reactions at the experimental temperature of 1003 K.

Reaction	∆G/KJ·mol^−1^
(2)	−119
(3)	476
(4)	−394
(5)	-

## Data Availability

The raw/processed data required to reproduce these findings cannot be shared at this time as the data also forms part of an ongoing study.
